# Crack Identification in CFRP Laminated Beams Using Multi-Resolution Modal Teager–Kaiser Energy under Noisy Environments

**DOI:** 10.3390/ma10060656

**Published:** 2017-06-15

**Authors:** Wei Xu, Maosen Cao, Keqin Ding, Maciej Radzieński, Wiesław Ostachowicz

**Affiliations:** 1 Department of Engineering Mechanics, Hohai University, Nanjing 210098, China; wxu@hhu.edu.cn; 2 Structural Health Monitoring Department, China Special Equipment Inspection and Research Institute, Beijing 100013, China; Dingkeqin@csei.org.cn; 3 Institute of Fluid-Flow Machinery, Polish Academy of Sciences, ul. Fiszera 14, Gdansk 80-231, Poland; mradzienski@imp.gda.pl (M.R.); wieslaw@imp.gda.pl (W.O.); 4 Faculty of Automotive and Construction Machinery, Warsaw University of Technology, Narbutta 84, Warsaw 02-524, Poland

**Keywords:** carbon fiber reinforced polymer laminated beam, crack identification, mode shape, Teager–Kaiser energy, multi-resolution modal Teager–Kaiser energy, scanning laser vibrometer

## Abstract

Carbon fiber reinforced polymer laminates are increasingly used in the aerospace and civil engineering fields. Identifying cracks in carbon fiber reinforced polymer laminated beam components is of considerable significance for ensuring the integrity and safety of the whole structures. With the development of high-resolution measurement technologies, mode-shape-based crack identification in such laminated beam components has become an active research focus. Despite its sensitivity to cracks, however, this method is susceptible to noise. To address this deficiency, this study proposes a new concept of multi-resolution modal Teager–Kaiser energy, which is the Teager–Kaiser energy of a mode shape represented in multi-resolution, for identifying cracks in carbon fiber reinforced polymer laminated beams. The efficacy of this concept is analytically demonstrated by identifying cracks in Timoshenko beams with general boundary conditions; and its applicability is validated by diagnosing cracks in a carbon fiber reinforced polymer laminated beam, whose mode shapes are precisely acquired via non-contact measurement using a scanning laser vibrometer. The analytical and experimental results show that multi-resolution modal Teager–Kaiser energy is capable of designating the presence and location of cracks in these beams under noisy environments. This proposed method holds promise for developing crack identification systems for carbon fiber reinforced polymer laminates.

## 1. Introduction

Carbon fiber reinforced polymer (CFRP) laminates are increasingly utilized in the aerospace and civil engineering fields for their low weight, high strength, and high stiffness [1,2,3]. During the long-term operation of CFRP laminated beam components, such as aircraft wings and wind turbine blades, cracks can occur on their surfaces, jeopardizing the integrity and safety of the whole structures [4,5,6]. Thus, identifying cracks in CFRP laminated beam is of great significance [7,8,9,10,11,12].

Commonly, physical properties such as strain [13,14], electrical resistance [15,16], and eddy current [17,18], can be utilized to identify cracks in CFRP laminates; changes in those physical properties can designate the presence and location of cracks. In contrast, dynamic characters, such as mode shapes, have lower capability to identify cracks in CFRP laminated beams because they are less sensitive to cracks than the aforementioned physical properties [19,20]. To tackle this deficiency, dynamic quantities such as modal curvature and modal strain energy were developed from the mode shape. Pandey et al. [21] proposed the modal curvature, which is the second-order derivative of a mode shape, to represent the crack-caused loss in bending stiffness, whereby cracks in beams can be identified by the change in the modal curvature. The modal curvature method has been further developed by many researchers in the last two decades [22,23,24,25,26,27,28,29,30,31,32,33,34]. The modal strain energy method is another method developed from mode shapes. Cracks can cause changes in modal strain energy, and in turn they can be identified by such changes [32,35,36,37,38,39]; recently, modern signal processing methods such as wavelet transform (WT) [40,41,42,43,44,45,46,47,48,49] and fractal dimension (FD) [50,51,52,53,54] have been applied to mode shapes for crack identification by characterizing the crack-caused singularities therein. Recent attention to crack identification methods relying on mode shape has focused on the robustness of the methods to environmental noise interference [30,33]. To precisely localize cracks, very small spatial sampling intervals matching the width of a crack are required, whereby noise components inevitably involved in densely-sampled mode shapes can cause intense noise interference, masking actual crack-caused changes [55,56]. Hence, developing noise-robust methods relying on mode shapes with the aim of precisely identifying cracks in CFRP laminated beams is the current research interest.

With this concern, this study proposes a physical concept modal Teager–Kaiser energy (M-TKE) derived from the mode shape. The M-TKE is the point-wise energy of a mode shape calculated by the Teager–Kaiser energy (TKE) operator, which features high sensitivity to structural damage. To enhance the noise robustness of the M-TKE, it is transformed to multi-resolution modal Teager–Kaiser energy (MRM-TKE) by the WT-based multi-resolution analysis (MRA) [57,58].

The rest of this paper is organized as follows. Section 2 introduces the fundamental theories of the MRA and the TKE operator. Section 3 proposes the M-TKE based on the TKE operator, and further develops it to the MRM-TKE by the MRA. Section 4 numerically proves the concept of the MRM-TKE to identify cracks in beams. Section 5 experimentally validates the applicability of the MRM-TKE to identification of cracks in CFRP laminated beams, whose modes shapes are precisely acquired via non-contact measurement using a scanning laser vibrometer (SLV). Section 6 presents the conclusions of this study.

## 2. Fundamental Theories

### 2.1. MRA

By the theory of the MRA [49,58], an orthonormal, compactly supported wavelet basis of space $L^{2}(\mathbb{R})$ of measureable, square integral functions is formed by dilating and translating a mother wavelet function ψ(x):

(1) $$\psi_{j,k}(x)=2^{-j/2}\psi (2^{-j}x-k);\;j,k\in \mathbb{Z},$$

where ℝ and ℤ denote sets of real and integer numbers, respectively. ψ(x) satisfies the following two-scale equation:

(2) $$\psi (x)=\sqrt{2}\sum_{k=0}^{M-1}g_{k}\phi (2x-k),$$

where ϕ(x) is a scaling function that is dilated and translated as

(3) $$\phi_{j,k}(x)=2^{-j/2}\phi (2^{-j}x-k);\;j,k\in \mathbb{Z}.$$

and ϕ(x) satisfies the following two-scale equation:

(4) $$\phi (x)=\sqrt{2}\sum_{k=0}^{M-1}h_{k}\phi (2x-k),$$

where ${\left\{g_{k}\right\}}_{k=0,...,M-1}$ and ${\left\{h_{k}\right\}}_{k=0,...,M-1}$ denote quadrature mirror filters and have the relationship:

(5) $$g_{k}={\left(-1\right)}^{k}h_{M-k-1};\;k=0,...,M-1.$$

Based on the orthonormal base expressed in Equation (1), the space $L^{2}(\mathbb{R})$ can be spanned by

(6) $$L^{2}(\mathbb{R})=\mathrm{span}(\psi_{j,k}:j,k\in \mathbb{Z}).$$

Equation (6) implies that the analysis and synthesis of an arbitrary signal f(x) in $L^{2}(\mathbb{R})$ can be, respectively, realized by

(7a) $$w_{j,k}=\int_{-\infty }^{\infty }f(x)\psi_{j,k}(x)dx,$$

(7b) $$f(x)=\sum_{j}\sum_{k}w_{j,k}\psi_{j,k}(x).$$

Such wavelets provide a framework for the MRA as stated in the following.

Derived from Equation (7), $W_{j}=\mathrm{span}(\psi_{j,k}:j,k\in \mathbb{Z})$ forms a subspace of $L^{2}(\mathbb{R})$, leading to

(8a) $$f_{j}=\sum_{k}w_{j,k}\psi_{j,k}(x),$$

(8b) $$f(x)=\sum_{j}f_{j}.$$

For all j, $W_{j}$ are orthogonal to each other, from which $L^{2}(\mathbb{R})$ is expressed as

(9) $$L^{2}(\mathbb{R})=\cdot \cdot \cdot ⊕W_{2}⊕W_{1}⊕W_{0}⊕W_{-1}⊕W_{-2}⊕\cdot \cdot \cdot ,$$

where ⊕ denotes summing vector spaces. On the other hand, $V_{j}=\mathrm{span}(\psi_{i,k}:i,k\in \mathbb{Z},i>j)$ forms a subspace of $L^{2}(\mathbb{R})$, which leads to $V_{j-1}=V_{j}⊕W_{j}$. Thus, $f_{j}$ in $V_{j}$ can be represented as

(10) $$f_{j}=\sum_{i>j}\sum_{k}w_{i,k}\psi_{i,k}(x)+\sum_{k}w_{j,k}\psi_{j,k}(x).$$

Substituting $V_{j}$ into Equation (9) results in

(11) $$L^{2}(\mathbb{R})=V_{j}⊕W_{j}⊕\cdot \cdot \cdot ⊕W_{1}⊕W_{0}⊕W_{-1}⊕W_{-2}⊕\cdot \cdot \cdot .$$

Thus, a sequence of closed subspace V nested as

(12) $$\cdot \cdot \cdot \subset V_{j+1}\subset V_{j}\subset \cdot \cdot \cdot \subset V_{1}\subset V_{0}\subset V_{-1}\subset \cdot \cdot \cdot ,$$

forms the MRA of $L^{2}(\mathbb{R})$.

Based on the above definitions, a signal f(x) in the subspace $V_{0}$ with the finest resolution can be decomposed into the first to the *N-*th level:

(13) $$f(x)=A_{N}(x)+\sum_{j=1}^{N}D_{j}(x),$$

where $A_{N}(x)$ is the approximation of f(x) at level N in $V_{N}$, and $D_{j}(x)$ is the detail of f(x) at level j in $W_{j}$. Equation (13) can be implemented by the discrete wavelet transform (DWT) [43]; the fundamental discrete wavelet, the Haar wavelet, is utilized for the MRA in this study. The scaling function ϕ(x) and mother wavelet function ψ(x) of the Haar wavelet are:

(14a) $$\phi (x)=\left\{ \begin{array}{ll} 1 & 0\leq x<1,\\ 0 & \mathrm{otherwise}, \end{array}\right.$$

(14b) $$\psi (x)=\left\{ \begin{array}{ll} 1 & 0\leq x<1/2,\\ -1 & 1/2\leq x\leq 1,\\ 0 & \mathrm{otherwise}, \end{array}\right.$$

whose quadrature mirror filters are

(15a) $${\left\{h_{k}\right\}}_{k=0,1}=\frac{1}{\sqrt{2}}\left[1,1\right],$$

(15b) $${\left\{g_{k}\right\}}_{k=0,1}=\frac{1}{\sqrt{2}}\left[1,-1\right].$$

### 2.2. TKE Operator

The TKE operator was proposed by Kaiser to measure the point-wise energy of a signal [57]. Let Y[p] be a discretized cosine signal:

(16) Y[p]=Ucos(Ωp+β),

where U is the amplitude, p is the sampling number, β is the initial phase, and Ω is the frequency specified by $\Omega =2\pi f/f_{s}$, with f being the analog frequency and $f_{s}$ the sampling frequency. The signal values at three successive points are:

(17) Y[p−1]=Ucos(Ω(p−1)+β), Y[p]=Ucos(Ωp+β), Y[p+1]=Ucos(Ω(p+1)+β).

According to the trigonometric identities, the signal values in Equation (17) have the following relationship:

(18) $$Y{\left[p\right]}^{2}-Y[p-1]Y[p+1]=U^{2}\sin^{2}(\Omega ).$$

Kaiser found that the left side of Equation (18) can be used to measure the point-wise energy of an oscillating signal, and this nonlinear operator is defined as the TKE operator, denoted Ψ(·) [57]:

(19) $$E[p]=\Psi \left(Y[p]\right)=Y^{2}[p]-Y[p-1]Y[p+1];$$

accordingly, the E[p] is called the TKE hereafter in this study.

**Figure 1:**
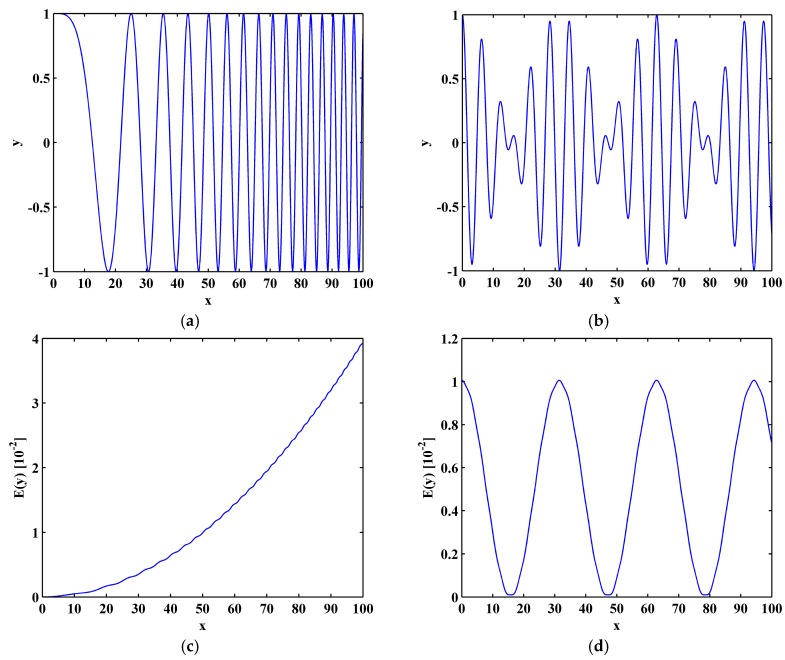
FM (**a**) and AM (**b**) signals; and their respective TKE (**c**,**d**).

The TKE operator is sensitive to change in the local frequency and amplitude of a signal [57]. To illustrate this property, consider a frequency-modulated (FM) signal (Figure 1a) and an amplitude-modulated (AM) signal y(x) (Figure 1b), whose Teager–Kaiser energies are calculated by Equation (19) and shown in Figure 1c,d, respectively. It can be seen that Teager–Kaiser energy can sensitively reflect changes in local frequency (Figure 1c) and amplitude (Figure 1d) in signals.

## 3. MRM-TKE for Identifying Cracks in Beams under Noisy Environments

This section proposes the M-TKE, from which the MRM-TKE, with stronger robustness to noise interference, is further developed by the WT-based MRA.

### 3.1. M-TKE

A mode shape of a Timoshenko beam can be expressed as [59]:

(20) $$W(x)=C_{1}\cosh\alpha_{1}x+C_{2}\sinh\alpha_{1}x+C_{3}\cos\alpha_{2}x+C_{4}\sin\alpha_{2}x,$$

where $C_{1}$, $C_{2}$, $C_{3}$, and $C_{4}$ are unknowns to be solved. $\alpha_{1}$ and $\alpha_{2}$ are parameters related to the natural frequency (the higher the natural frequency is, the lager are $\alpha_{1}$ and $\alpha_{2}$). For a high-order mode shape, Equation (20) can be further written as [60,61]:

(21) $$W(x)={\bar{C}}_{1}e^{-\alpha_{1}x}+{\bar{C}}_{2}e^{-\alpha_{1}(L-x)}+C_{3}\cos\alpha_{2}x+C_{4}\sin\alpha_{2}x,$$

where L is the beam length. W(x) in Equation (21) can be divided into two terms, the decaying term $W_{D}(x)$ and the steady-state term $W_{S}(x)$:

(22a) $$W_{D}(x)={\bar{C}}_{1}e^{-\alpha_{1}x}+{\bar{C}}_{2}e^{-\alpha_{1}(L-x)},$$

(22b) $$W_{S}(x)=C_{3}\cos\alpha_{2}x+C_{4}\sin\alpha_{2}x.$$

The value of the decaying term $W_{D}(x)$ exponentially decays from the boundaries at x=0 and x=L. The distance from boundaries to the locations where $W_{D}(x)$ approximates zero is defined as the boundary-effect interval, denoted as d. As per Equation (22a), the higher the mode order is, the larger is $\alpha_{1}$, and then the smaller are the boundary-effect intervals. Outside the boundary-effect intervals, $W_{S}(x)$ dominates the high-order mode shape component, $W_{D}(x)$ contributes little to it. Hence, such high-order mode shape components can be approximately represented as $W_{S}(x)$:

(23) $$W(x)\approx W_{S}(x)=C\cos(\alpha_{2}x+\beta );\;x\in [d,L-d].$$

where $C=\sqrt{{C_{3}}^{2}+{C_{4}}^{2}}$, $\tan\beta =-\frac{C_{4}}{C_{3}}$. The discrete form of W(x) in Equation (23) can be written as:

(24) W[x]≈Ccos(Ωx+β);  x∈[d,L−d].

where $\Omega =\alpha_{2}/f_{s}$ with $f_{s}$ being the spatial sampling frequency. The TKE of the mode shape W[x], namely the M-TKE, can be calculated by Equation (17):

(25) $$E(W[x])=\Psi (W[x])=W{\left[x\right]}^{2}-W[x-1]W[x+1];\;x\in [d,L-d].$$

By Equation (18), E(W[x]) approximates a constant of $C^{2}\sin^{2}\Omega$:

(26) $$E(W[x])\approx \Psi (C\cos(\Omega x+\beta ))=C^{2}\sin^{2}\Omega ;\;x\in [d,L-d].$$

Owing to the sensitivity of the TKE operator to changes in the local frequency and amplitude of signals (shown in Figure 1), the M-TKE can be sensitive to slight changes in C and Ω caused by cracks, whereby cracks can be clearly identified by such changes.

### 3.2. MRM-TKE

Noise components are inevitably involved in measured mode shapes, and Kaiser has proved that the TKE operator is very prone to noise interference [57]; therefore, the vulnerability of the M-TKE to noise interference can hamper its applicability in identifying cracks under noisy environments. To overcome this deficiency, the M-TKE is ameliorated by the WT-based MRA, whereby the MRM-TKE has stronger noise robustness. In accordance with the MRA introduced in Section 2, W[x] can be decomposed into the *N-*th approximation $A_{N}[x]$ and the first to *j*-th details $D_{j}[x]$ (j=1,...,N) by Equation (13), and the M-TKE can be expressed as:

(27) $$E(W[x])=\Psi (W[x])=\Psi (A_{N}[x])+\sum_{j=1}^{N}D_{j}[x]);\;x\in [d,L-d].$$

By discarding the details $D_{j}[x]$ up to a satisficing level N that contain noise components and substituting the retained approximation $A_{N}[x]$ that contains damage features for W[x] in Equation (25), the MRM-TKE is defined, denoted as $E_{N}(W[x])$:

(28) $$E_{N}(W[x])=E(A_{N}[x])=A_{N}{\left[x\right]}^{2}-A_{N}[x-1]A_{N}[x+1];\;x\in [d,L-d].$$

In contrast to the M-TKE with one-fold resolution, the resolution of the MRM-TKE is adjustable by the level N, whereby noise components in the M-TKE can be eliminated at a satisficing level in the MRM-TKE; synchronously, crack features in the MRM-TKE can be retained for crack identification. It is worth mentioning that the MRM-TKE method is a non-baseline method, requiring no structural baseline information such as temperature, materials, geometry, and boundary conditions.

## 4. Proof of Concept

Without loss of generality of the MRM-TKE method, the concept of the MRM-TKE to identify cracks is proven on beams of general materials and boundary conditions with emphasis on its noise robustness.

### 4.1. Free Vibration of Timoshenko Beams with Cracks

Cracks in a beam are modeled as linear rotational springs with the bending constant of each crack determined by the fracture mechanics principle [62,63]:

(29) K=1/c,  c=(5.346H/EI)J(ξ),

where E is the Young’s modulus, I is the moment of inertia, K is the bending constant of the spring, H is the beam thickness, ξ=a/H is the crack depth ratio with a the crack depth, and J(ξ) is the dimensionless local compliance function:

(30) $$\begin{array}{l} J(\xi )=1.8624{\left(\xi \right)}^{2}-3.95{\left(\xi \right)}^{3}+16.375{\left(\xi \right)}^{4}-37.226{\left(\xi \right)}^{5}\\ \;+76.81{\left(\xi \right)}^{6}-126.9{\left(\xi \right)}^{7}+172{\left(\xi \right)}^{8}-143.97{\left(\xi \right)}^{9}+66.56{\left(\xi \right)}^{10}. \end{array}$$

**Figure 2:**
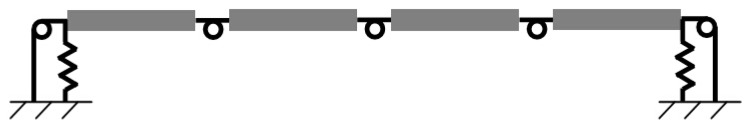
Analytical model of *n*-crack beam with *n* being 3.

As illustrated in Figure 2, the beam is divided into n+1 segments by n cracks with each adjacent pair of segments being linked by a crack.

According to the theory of Timoshenko beams, governing equations for the flexural vibration of the *i-*th beam segment are [59]:

(31a) $$EI\frac{\partial^{2}\varphi_{i}(x,t)}{\partial x^{2}}+kGA\left(\frac{\partial w_{i}(x,t)}{\partial x}-\varphi_{i}(x,t)\right)-\rho I\frac{\partial^{2}\varphi_{i}(x,t)}{\partial t^{2}}=0,$$

(31b) $$kG\left(\frac{\partial^{2}w_{i}(x,t)}{\partial x^{2}}-\frac{\partial \varphi (x_{i},t)}{\partial x}\right)-\rho \frac{\partial^{2}w(x_{i},t)}{\partial t^{2}}=0,$$

where $w_{i}(x,t)$ is the transverse deflection; $\varphi_{i}(x,t)$ is the slope of deflection due to the bending; and E, G, I, ρ, A, and k are the Young’s modulus, shear modulus, moment of inertia, material density, cross-sectional area, and the shear coefficient for the cross-section, respectively.

Solutions to Equation (31) consist of spatial and temporal parts:

(32a) $$w_{i}(x,t)=LW_{i}(x)e^{j\omega t},$$

(32b) $$\varphi_{i}(x,t)=\theta_{i}(x)e^{j\omega t},$$

where ω is the angular frequency of vibration, j is the imaginary unit, and $W_{i}$ and $\theta_{i}$ are the amplitudes of transverse deflection and rotational angle of the *i-*th beam segment, respectively. Substituting Equation (32) into Equation (31), with the coordinate variables $\zeta =\frac{x}{L}$ and $\varsigma =\frac{t}{\sqrt{L}}$, geometric and material variables $\vartheta =\frac{E}{kG}$, $r=\frac{1}{AL^{2}}$, s=ϑr, $\tau =\frac{\rho A}{EI}L^{4}\omega^{2}$, $a=\frac{\tau (r+s)}{2}$ and b=τ(τrs−1), yields the equations [64]:

(33a) $$\frac{d^{4}}{d\zeta^{4}}W_{i}(\zeta )+2a\frac{d^{2}}{d\zeta^{2}}W_{i}(\zeta )+bW_{i}(\zeta )=0,$$

(33b) $$\frac{d^{4}}{d\zeta^{4}}\theta_{i}(\zeta )+2a\frac{d^{2}}{d\zeta^{2}}\theta_{i}(\zeta )+b\theta_{i}(\zeta )=0.$$

Let $\gamma_{1}={\left(\sqrt{a^{2}-b}-a\right)}^{1/2}$, $\gamma_{2}={\left(\sqrt{a^{2}-b}+a\right)}^{1/2}$, $m_{1}=\frac{\tau s+\gamma_{1}^{2}}{\gamma_{1}}$, $m_{2}=\frac{\tau s-\gamma_{2}^{2}}{\gamma_{2}}$, the solutions of $W_{i}$ and $\theta_{i}$ can be expressed as [65,66,67]:

(34a) $$W_{i}(\zeta )=C_{i1}\cosh\gamma_{1}\zeta_{i}+C_{i2}\sinh\gamma_{1}\zeta_{i}+C_{i3}\cos\gamma_{2}\zeta_{i}+C_{i4}\sin\gamma_{2}\zeta_{i},$$

(34b) $$\theta_{i}(\zeta )=C_{i1}m_{1}\sinh\gamma_{1}\zeta_{i}+C_{i2}m_{1}\cosh\gamma_{1}\zeta_{i}+C_{i3}m_{2}\sin\gamma_{2}\zeta_{i}-C_{i4}m_{2}\cos\gamma_{2}\zeta_{i}.$$

Compatible conditions of displacement, slope, moment, and shear force in the crack locations $\zeta_{i}$ can be expressed as [67]:

(35) $${W_{i}=W_{i+1}|}_{\zeta =\zeta_{i}},\;{W_{i}^{\prime }-W_{i+1}^{\prime }=-\frac{EI}{KL}\theta_{i}^{\prime }|}_{\zeta =\zeta_{i}},\;{\theta_{i}^{\prime }=\theta_{i+1}^{\prime }|}_{\zeta =\zeta_{i}},\;{W_{i}^{\prime }-\theta_{i}=W_{i+1}^{\prime }-\theta_{i+1}|}_{\zeta =\zeta_{i}}.$$

Without loss of generality, boundaries at each end of the beam are simulated by a pair of linear springs, by which the boundary conditions at each end can be expressed as [68]:

(36a) $$WLK_{T}-AGk(W^{\prime }-\theta )=0,$$

(36b) $$\theta LK_{S}-EI\theta^{\prime }=0,$$

where $K_{T}$ and $K_{S}$ are constants of springs providing translational and rotational restraints, respectively. Equation (36) can be further written as [68]:

(37a) $$W=\eta_{T}(W^{\prime }-\theta ),$$

(37b) $$\theta =\eta_{S}\theta^{\prime },$$

where parameters $\eta_{T}=\frac{AGk}{LK_{T}}$ and $\eta_{S}=\frac{EI}{LK_{S}}$. According to Equation (37), four common boundary conditions can be represented: for a simply supported (SS) end, $\eta_{T}=0$ and $\eta_{S}=\infty$ produce boundary conditions with W=0 and $\theta^{\prime }=0$; for a free (F) end, $\eta_{T}=\infty$ and $\eta_{S}=\infty$ produce boundary conditions with $W^{\prime }-\theta =0$ and $\theta^{\prime }=0$; for a free-shear (FS) end, $\eta_{T}=\infty$ and $\eta_{S}=0$ produce boundary conditions with $W^{\prime }-\theta =0$ and θ=0; and for a clamped (C) end, $\eta_{T}=0$ and $\eta_{S}=0$ produce boundary conditions with W=0 and θ=0.

Substituting Equation (34) into the four equations of boundary conditions at the two ends and 4n equations of compatible conditions at the crack locations, a group of simultaneous equations with respect to ω can be obtained:

(38) D(ω)C=0,

where C is a column vector of $C_{i1}$, $C_{i2}$, $C_{i3}$ and $C_{i4}$ (i=1,2,⋯,n+1), and D is a 4(n+1)×4(n+1) matrix. To achieve nontrivial solutions, the determinant of D, D(ω), is set to zero to produce the frequency equation:

(39) D(ω)=0.

Solving Equation (39) produces a sequence of natural frequencies $\omega_{m}$ [69]; provided with $\omega_{m}$, the corresponding coefficient vector $C_{m}$ can be derived from Equation (38); substituting $\omega_{m}$ and $C_{m}$ into Equation (34a), the *m*-th mode shape can be obtained.

### 4.2. Crack Identification

A general beam with the dimensions of length 500 mm, width 30 mm, and depth 10 mm is taken as a specimen. Three cracks are introduced at locations *x* = 125 mm ($\zeta_{1}=0.25$), 275 mm ($\zeta_{2}=0.55$), and 375 mm ($\zeta_{3}=0.75$), with depths of 2.5 mm ($\xi_{1}=0.25$), 2 mm ($\xi_{2}=0.2$), and 3 mm ($\xi_{3}=0.3$), respectively. Three scenarios associated with three common types of boundary conditions are considered: SS-SS, C-F, and C-SF boundary conditions for the sixth, seventh, and eighth modes, respectively.

**Figure 3:**
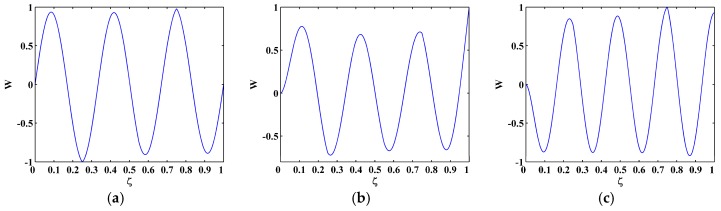
(**a**) Sixth; (**b**) seventh; and (**c**) eighth mode shapes of SS-SS, C-F, and C-FS beams.

**Figure 4:**
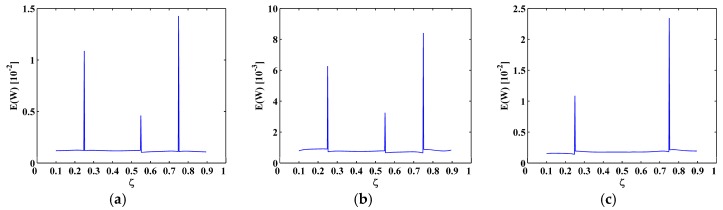
M-TKE for the (**a**) sixth; (**b**) seventh; and (**c**) eighth noise-free mode shapes.

**Figure 5:**
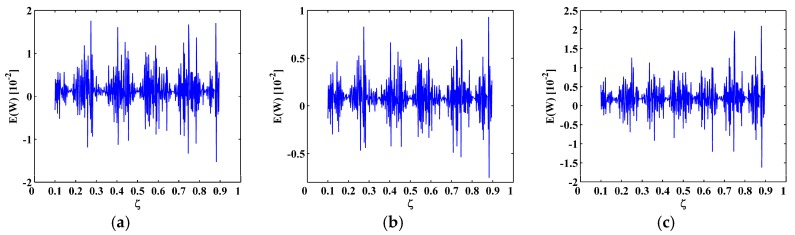
M-TKE for the (**a**) sixth; (**b**) seventh; and (**c**) eighth mode shapes with 60 dB SNR.

The sixth, seventh and eighth sampled mode shapes W[ζ] associated with the SS-SS, C-F, and C-FS boundary conditions (Figure 3) are produced following the procedure given in Section 4.1 with 501 uniformly distributed sampling points. The corresponding M-TKE E(W[ζ]) is obtained by Equation (25), and shown in Figure 4 with values in boundary-effect interval (d=0.1) vanished. It can be seen in Figure 4a,b that three peaks in E(W[ζ]) evidently indicate the presence of three cracks and clearly pinpoint the cracks at ζ=0.25, 0.55, and 0.75, which correspond to the actual crack locations $\zeta_{1}=0.25$, $\zeta_{2}=0.55$, and $\zeta_{3}=0.75$; in Figure 4c, only two peaks appear at $\zeta_{1}=0.25$ and $\zeta_{3}=0.75$ because the location of the second crack at $\zeta_{2}=0.55$ is close to one of the nodes, and it is hard to identify such cracks because vibration near a node is always of close-to-zero amplitude [27,70]. Thus, under a noise-free environment, the M-TKE is capable of identifying cracks in beams. For actually measured mode shapes, however, noise components are inevitably incorporated. To simulate a normal noisy environment, white Gaussian noise is added to the W[ζ] to produce noisy mode shapes of 60 dB signal-to-noise ratio (SNR) [33]; the lower the SNR is, the noisier is the mode shape. The corresponding noise-contaminated E(W[ζ]) is obtained and shown in Figure 5, where intense noise interference considerably masks crack-caused peaks in the E(W[ζ]). Thus, susceptibility to noise severely hampers the capability of the M-TKE to identify cracks in beams under noisy environments.

**Figure 6:**
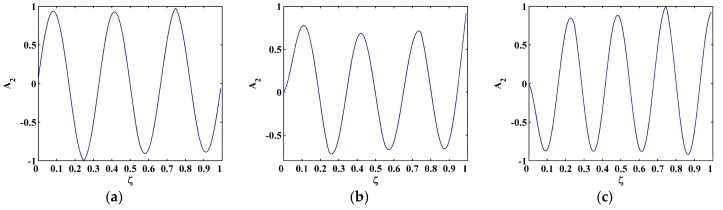
The second-level approximations of the (**a**) sixth; (**b**) seventh; and (**c**) eighth mode shapes with 60 dB SNR.

**Figure 7:**
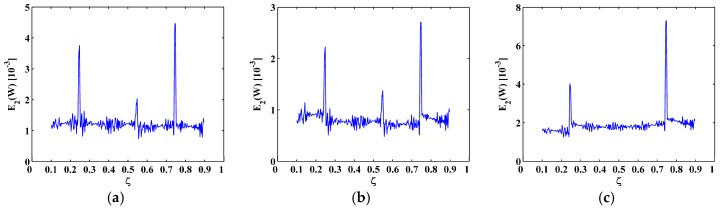
MRM-TKE for the (**a**) sixth; (**b**) seventh; and (**c**) eighth mode shapes with 60 dB SNR.

To eliminate noise interference, the second-level (N=2) approximations $A_{2}[\zeta ]$ are extracted from the mode shapes W[ζ] by Equation (13), as shown in Figure 6; then the MRM-TKE $E_{2}(W[\zeta ])$ is obtained by Equation (28), as shown in Figure 7 with values in the boundary-effect interval (d=0.1) vanished. It can be seen from Figure 7 that noise interference is basically eliminated and crack-caused peaks can be clearly identified. In Figure 7a,b, three damage-induced peaks stand out obviously and clearly pinpoint the cracks at ζ=0.25, 0.55, and 0.75, which correspond to the actual crack locations $\zeta_{1}=0.25$, $\zeta_{2}=0.55$, and $\zeta_{3}=0.75$; in Figure 7c only two peaks appear at $\zeta_{1}=0.25$ and $\zeta_{3}=0.75$ because of the node effect mentioned before. Thus, demonstrably superior to the M-TKE, the MRM-TKE features much stronger robustness to noise interference and is capable of identifying cracks in beams under noisy environments.

### 4.3. Noise Tolerance

**Figure 8:**
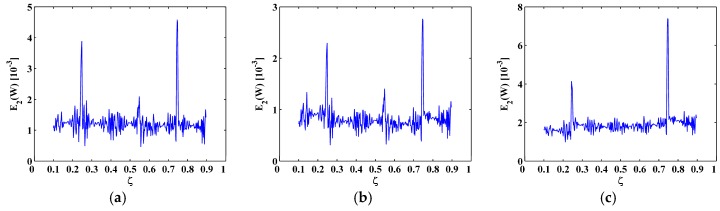
MRM-TKE for the (**a**) sixth; (**b**) seventh; and (**c**) eighth mode shapes with 55 dB SNR.

**Figure 9:**
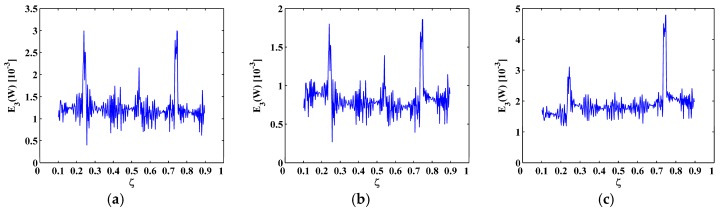
MRM-TKE for the (**a**) sixth; (**b**) seventh; and (**c**) eighth mode shapes with 50 dB SNR.

**Figure 10:**
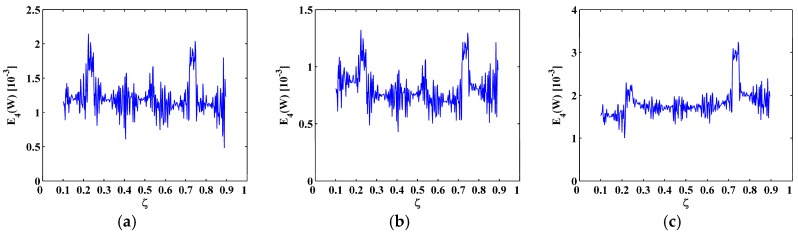
MRM-TKE for the (**a**) sixth; (**b**) seventh; and (**c**) eighth mode shapes with 45 dB SNR.

To demonstrate the noise tolerance of the MRM-TKE, a broader range of noise levels with decreasing SNRs of 55 dB, 50 dB, and 45 dB are considered; the corresponding results are shown in Figure 8, Figure 9 and Figure 10, respectively. At the noise level of 55 dB SNR, the MRM-TKE appears slightly noisier than the results for the 60 dB SNR (Figure 7), and the crack-caused peaks can still clearly identify the three cracks in the SS-SS beam and the C-F beam (Figure 8a,b), and the first and the third cracks in the C-FS beam (Figure 8c); at the noise level of 50 dB SNR, noise interference becomes more intense, but peaks can be still identified (Figure 9) by increasing the level of the approximation to N=3; at the noise level of 45 dB SNR, noise interference becomes severe, and peaks are just distinguishable (Figure 10) when the level of the approximation is further increased to N=4. Thus, noise level of 45 dB SNR can be regarded as the limit of the noise tolerance of the MRM-TKE for the given scenarios.

**Figure 11:**
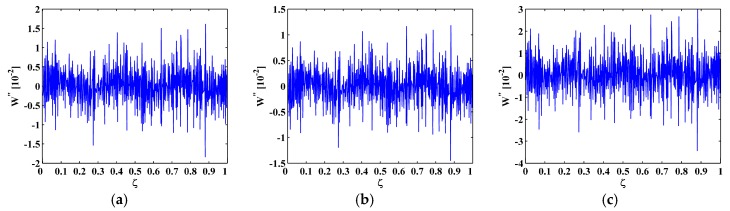
Modal curvatures for the (**a**) sixth; (**b**) seventh; and (**c**) eighth mode shapes with 45 dB SNR.

Modal curvature method [21], one of the most commonly used methods for crack identification in beams, is employed for comparison. Modal curvatures at the limit noise level of 45 dB are shown in Figure 11. It can be seen in Figure 11 that similar to the results of the M-TKE (Figure 5), damage features can be barely identified due to intense noise interference. Thus, MRM-TKE features stronger noise robustness than the commonly utilized modal curvature for crack identification in beams.

## 5. Experimental Validation

The applicability of the MRM-TKE to the identification of cracks is experimentally validated on a CFRP laminated beam, whose modes shapes are acquired via non-contact measurement using a SLV.

### 5.1. Setup

**Figure 12:**
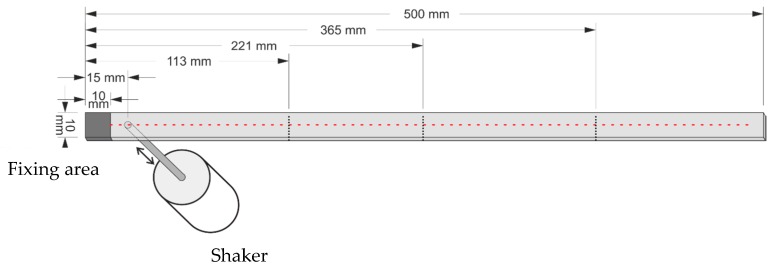
Dimensions in millimeters of cracked beam with shaker and measurement points.

A CFRP laminated beam of length 500 mm, width 10 mm, and depth 1.5 mm, consisting of five plies each 0.3 mm in thickness, is taken as an experimental specimen. The dimensions of the beam are shown in Figure 12 in millimeters. As shown in Figure 12, the beam is clamped at the left end with the fixing area spanning 10 mm from the left edge. Four damage scenarios, Scenarios I, II, III and IV, are considered. First, three through-width cracks are manufactured in the first two plies on the surface, about 0.5 mm (ξ=1/3) in depth between the first and the second plies. In Scenario I, the fourth mode is considered; in Scenario II, the fifth mode is considered. Then, all three cracks are increased to about 0.9 mm (ξ=0.6) in depth through the third ply. In Scenario III, the fourth mode is considered and, in Scenario IV, the fifth mode is considered. The first, second, and third cracks, indicated by dashed lines in Figure 12, occur at locations 113 mm, 221 mm, and 365 mm from the left edge, respectively.

**Figure 13:**
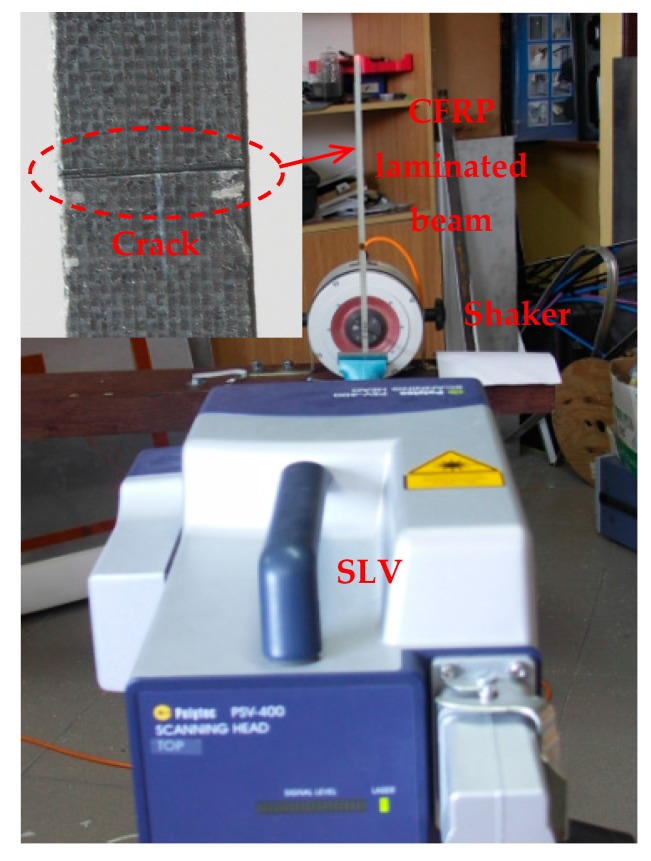
Experimental setup.

A vibration shaker (4809, B&K, Nærum, Denmark), attached to the cracked side of the beam, 15 mm from its left edge, acts as an actuator to excite the beam. When the beam vibrates under harmonic excitation at the fourth natural frequency of 241.79 Hz, the SLV (PSV-400, Polytec, Waldbronn, Germany) is used as a sensor to scan the intact side of the beam to acquire the operating deflection shape (ODS), that can be regarded as the fourth mode shape for this lightly-damped beam [71]. The SLV scans over 499 measurement points uniformly distributed on the intact surface, 10 mm through 496 mm from the left edge; the dimensionless locations for the first, second, and third cracks in the scanning length are $\zeta_{1}=0.212$, $\zeta_{2}=0.434$, and $\zeta_{3}=0.730$, respectively. Figure 13 shows the experimental setup including the SLV and the shaker along with a zoomed-in view of the crack in the CFRP laminated beam.

### 5.2. Experimental Results

**Figure 14:**
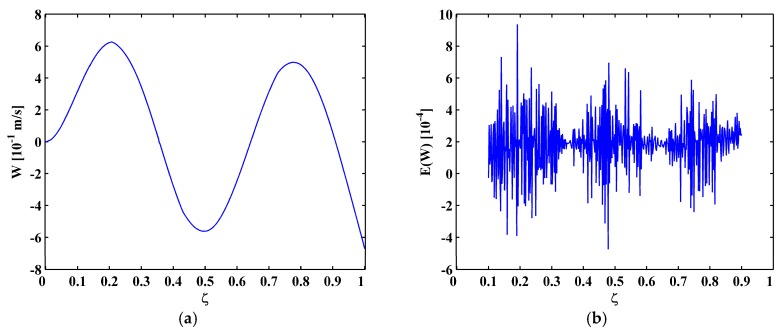
The fourth mode shape (**a**) and M-TKE (**b**) for Scenario I.

**Figure 15:**
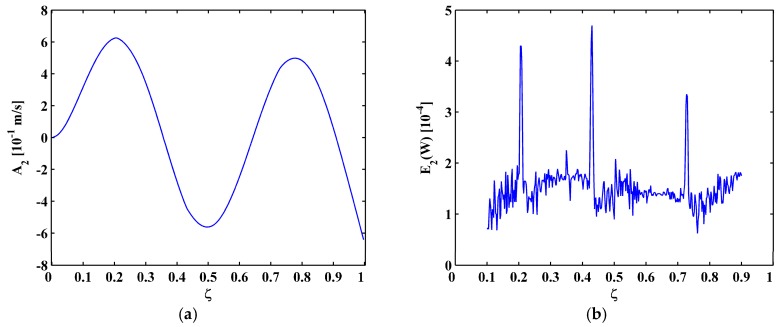
Second-level approximation of the fourth mode shape (**a**) and MRM-TKE (**b**) for Scenario I.

In Scenario I, the fourth mode shape W[ζ] is shown in Figure 14a, from which the M-TKE E(W[ζ]) is obtained by Equation (25) and is shown in Figure 14b. In Figure 14b, damage features of E(W[ζ]) can be barely identified due to intense noise interference. The second-level (N=2) approximation $A_{N}[\zeta ]$ is obtained by Equation (13), and is shown in Figure 15a, from which the MRM-TKE $E_{N}(W[\zeta ])$ is obtained by Equation (28) and is shown in Figure 15b, where three crack-caused peaks stand out obviously, clearly pinpointing three cracks at about $\zeta_{1}=0.21$, $\zeta_{2}=0.435$, and $\zeta_{3}=0.73$, agreeing well with the actual locations of the first, second, and third cracks.

**Figure 16:**
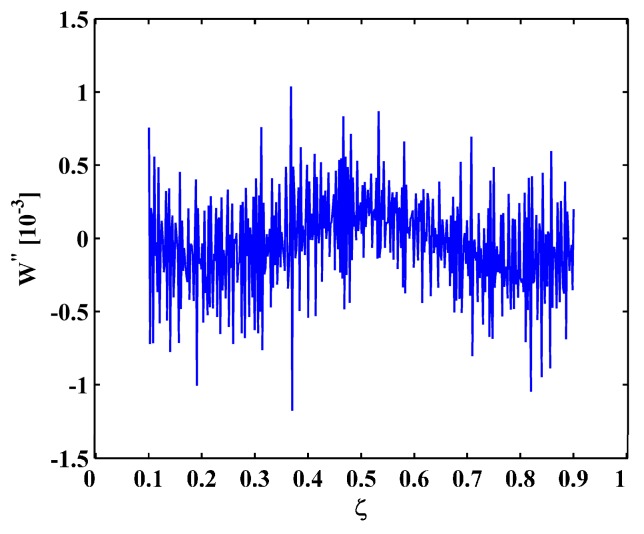
Modal curvature for Scenario I.

The results of the experiment validate the contention that the M-TKE lacks noise robustness, whereas the MRM-TKE is robust to noise interference, capable of designating the presence and location of cracks in CFRP laminated beams under noisy environments. Modal curvature for Scenario I is shown in Figure 16 for comparison. It can be seen in Figure 16 that, similar to the analytical results in Figure 11, damage features can be barely identified due to intense noise interference.

**Figure 17:**
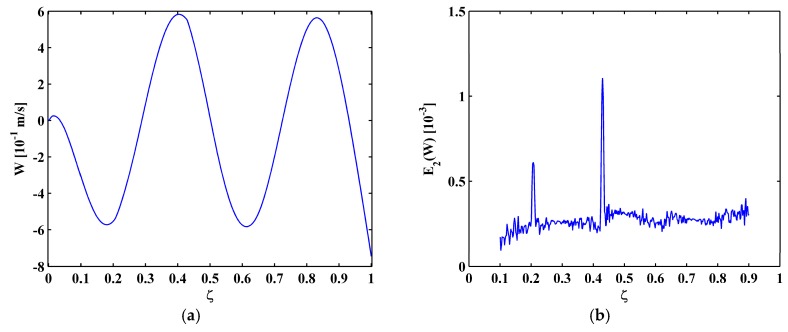
The fifth mode shape (**a**) and MRM-TKE (**b**) for Scenario II.

**Figure 18:**
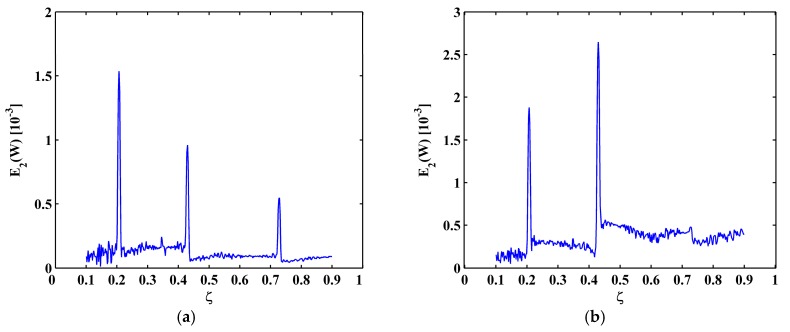
MRM-TKE for Scenario III (**a**) and Scenario IV (**b**).

For Scenarios II, the fifth mode shape is shown in Figure 17a, and the corresponding MRM-TKE $E_{2}(W)$ is shown in Figure 17b. In Figure 17b, only two peaks appear at $\zeta_{1}=0.21$ and $\zeta_{2}=0.435$, because the third crack at $\zeta_{3}=0.73$ is close to one of the nodes. The reason has been given in Section 4.2 that vibration close to a node is always of close-to-zero amplitude. For Scenarios III and IV with deeper cracks, crack-caused peaks are of larger amplitudes and more evident, which is more beneficial to crack identification. For Scenario III, three peaks clearly identify all three cracks (Figure 18a); and, for Scenario IV, only the first and third cracks can be identified (Figure 18b).

It can be seen from the MRM-TKE for Scenarios I to IV that deeper cracks are more evident to be identified by the MRM-TKE because the peak of the MRM-TKE increases with crack depth; cracks close to nodes are hard to identify because of the node effect. In addition, when smaller depths of cracks, e.g., 0.25 mm, are considered, the peaks in the MRM-TKE become less prominent.

## 6. Conclusions

To identify cracks in CFRP laminated beams under noisy environments, this study proposes a physical concept M-TKE derived from a mode shape, that is sensitive to crack-caused changes in the local frequency and amplitude. To enhance the noise robustness of the M-TKE, it is transformed to the MRM-TKE by the WT-based MRA. The efficacy of this concept is analytically demonstrated by identifying cracks in Timoshenko beams with general boundary conditions; and its applicability is validated in a CFRP laminated beam, whose mode shapes are precisely acquired via non-contact measurement using a SLV. The analytical and experimental results show that the MRM-TKE is capable of designating the presence and location of cracks in CFRP laminated beams under noisy environments. Some conclusions are drawn below:

1. Cracks can cause rapid changes in the M-TKE because the TKE operator is sensitive to slight changes in the local frequency and amplitude of a mode shape. However, the M-TKE is very prone to noise interference and therefore lacks the robustness to identify cracks under noise environments.
2. To enhance the noise robustness of the M-TKE, the MRM-TKE is developed from the M-TKE with the WT-based MRA, whereby noise components in the M-TKE can be eliminated at a satisficing level in the MRM-TKE; synchronously, damage-caused change in the M-TKE can be retained in the MRM-TKE. Thus, the MRM-TKE is capable of identifying cracks in CFRP laminated beams under noisy environments.
3. The MRM-TKE method is a non-baseline method, requiring no structural baseline information such as temperature, materials, geometry, and boundary conditions. The only requirement for the MRM-TKE method is that high-order modes are needed for small boundary-effect intervals.
